# Review of robotic surgery platforms and end effectors

**DOI:** 10.1007/s11701-023-01781-x

**Published:** 2024-02-13

**Authors:** Francesco Cepolina, Roberto Razzoli

**Affiliations:** DIMEC-PMAR Lab, Instrumental Robot Design Research Group, Department of Machines Mechanics and Design, University of Genova, Via All’Opera Pia 15A, 16145 Genoa, Italy

**Keywords:** Robotic surgery, Robotically assisted, Surgical robotics, Endoscopic, End effectors, Commercial products

## Abstract

In the last 50 years, the number of companies producing automated devices for surgical operations has grown extensively. The population started to be more confident about the technology capabilities. The first patents related to surgical robotics are expiring and this knowledge is becoming a common base for the development of future surgical robotics. The review describes some of the most popular companies manufacturing surgical robots. The list of the company does not pretend to be exhaustive but wishes to give an overview of the sector. Due to space constraints, only a limited selction of companies is reported. Most of the companies described are born in America or Europe. Advantages and limitations of each product firm are described. A special focus is given to the end effectors; their shape and dexterity are crucial for the positive outcome of the surgical operations. New robots are developed every year, and existing robots are allowed to perform a wider range of procedures. Robotic technologies improve the abilities of surgeons in the domains of urology, gynecology, neurology, spine surgery, orthopedic reconstruction (knee, shoulder), hair restoration, oral surgery, thoracic surgery, laparoscopic surgery, and endoscopy.

## Introduction

The advantages of robotic surgery in terms of safety, precision, and accuracy have made it more and more popular in recent years [1]. Today the market is offering a wide range of surgical robots. The competition among surgical robot producers helps us to raise standards and improve their value/cost ratio. The review aims to describe the robotics devices produced by some of the most popular manufacturing companies. Special attention is devoted to the end effectors, key components essential for performing the surgical task [2, 3]. The dexterity of end effectors is crucial for the correct execution of surgical procedures. End-effectors have the task of manipulating tissues and instruments. They can have small dimensions and an extremely fine tip, to reach and grip tissues that sometimes are very delicate. Reusable end effectors can be built using titanium or stainless steel, which are resistant to corrosion and degradation. Disposables are often made of 304 stainless steel, while reusable of 316L or equivalent. Both 304 and 316L are suitable for pressing and sintering in metal injection molding (MIM), while 316L has the highest level of corrosion resistance and is stronger at high temperatures. Abrasive materials allow the tip of the end effectors to have a better grasp.

The most common shapes of end effectors are:

*Maryland*: these are used to grasp and manipulate delicate tissues, such as blood vessels and nerves.

*Curved*: these are used to reach hard-to-access areas.

*Traumatic*: they have a claw-shaped tip, which allows you to grip and hold tissues. They are used to manipulate delicate tissues, such as the skin or blood vessels.

*Fenestrated*: they have a sharp tip with a set of teeth, allowing you to grip and hold tissues more securely. They are used to manipulate thicker tissues, such as muscles or tendons.

*Pre-tensioned needle holders*: they are used to suture tissues or to grasp needles and other instruments.

Microsurgery end effectors are used in a variety of surgeries, including:

*Plastic surgery*: for the correction of cosmetic imperfections or the reconstruction of damaged tissues.

*Orthopedic surgery*: for the repair of bones, joints, or tendons.

*Ophthalmic surgery*: for the correction of visual defects or the removal of cataracts.

*Dental surgery*: for the removal of cavities or the implantation of artificial teeth.

*Neurosurgery*: to treat nervous diseases like brain tumors.

Microsurgery end effectors are essential tools for precision surgery. Thanks to their extremely fine tip and robust construction, they allow you to perform delicate and complex surgeries with the utmost precision.

## Methods

Open surgery, compared to minimally invasive surgery, allows more freedom of movement, and better feedback of the scene. Patients subjected to a minimally invasive approach enjoy shorter recovery time (smaller incisions), less infection risk, lower pain, and less blood loss. The minimally invasive approach is relatively expensive in terms of equipment cost, setup time and surgeon training. Surgeons operating with minimally invasive robots suffer less fatigue but may be more stressed, due to the loss of direct contact with the body of the patient. Hands tremor filtering, view magnifications and motion scaling are nice features that raise the price of minimally robotic surgery compared to minimally invasive surgery.

The dexterity of robotic surgery tools is high, while their control can be less intuitive/direct than the handheld laparoscopic surgery tools. Minimally invasive robotic surgery allows the best precision and the ability to access remote places inside the human body.

The objective of the research is to collect, in a single place, a selection of surgical robots. Due to space constraints, only a few robots are recalled. For each platform, the main features are mentioned and compared. Robots' accuracy and sensitivity are crucial. A discussion about disposable, reusable and reposable instruments is reported: there is a trade-off between the number of usages and instruments cost. Most advanced robots feature tremor control and Stereo-optics.

To enhance the instrument’s dexterity a wrist or a snake articulation is provided.

### Commercial products

This section of the review examines the offerings of some of the most well-known manufacturers of surgical robots. The choice of the right surgical platform is not easy. Several aspects need to be considered. Each product has its characteristics. Each robot can perform specific surgical procedures.

Moreover, according to the size and dexterity of the end effector, the same procedure may be executed differently. The accuracy of the execution of the task is also a specific characteristic to consider. Patient safety is always of paramount importance; safety measures can vary among surgical platforms. There is always an important trade-off between the device’s capabilities and the time the surgeons need to learn and operate. The level of training and support offered is important to optimize the surgeon’s learning curve [4–6]. The surgical platforms may have different purchase and operating costs. System versatility and availability are also important; some systems may be available only in certain regions while others may be suitable only for a limited set of procedures—statistics on surgical operation success rate and post-recovery time help to consider the patient benefit. According to our perspective, user and patient feedback can be used best to assess the performance of a surgical platform. For each firm considered, location and foundation date are reported (Table 1). Some companies were founded many years ago while their first surgical robots were more recently developed.

**Table 1:** Selection of Surgical Robot Producers

Company Name	Headquarters	Company founded	Field of operation
Accuray Incorporated	United States	1990	Stereotactic radiation therapy, radiation therapy, robotic-assisted radiosurgery
Asensus Surgical	United States	2006	Laparoscopic surgery
Auris Health Inc.	United States	2007	Bronchoscopy, urological surgery
AVRA Medical Robotics Inc.	Canada	2015	Laparoscopy
BEC Medical	Germany	2003	Radiotherapy, biopsy
Brainlab	Germany	1989	Image guided surgery, spine surgery
Cambridge Medical Robotics Ltd	United Kingdom	2014	Minimally invasive surgery
Collin medical	France	1820	Ear surgery
Corindus Vascular Robotics Inc.	United States	2002	Vascular surgery
Distalmotion	Switzerland	2012	Minimally invasive surgery
EndoControl SAS	France	2006	Endoscopy
EndoMaster Surgical System	South Korea	2011	Laparoscopy
Human Xtensions	Israel	2012	Hand held laparoscopy
Intuitive Surgical Inc.	United States	1995	Minimally invasive surgery
Mazor Robotics Ltd	Israel	2001	Spine surgery
Medbot	China	2014	Laparoscopy
Medrobotics Corporation	United States	2005	Laparoscopy
Medtronic Inc.	Ireland (U.S. tax inversion)	1949	Spine surgery
Meerecompany Inc.	United States	1984	Laparoscopy
Microbot Medical Inc.	United States	2010	Neurosurgery
MMI	Italy	2015	Neurosurgery, orthopedic surgery, microsurgery
Myomo Inc.	United States	2004	Orthotics
Microsure	Netherlands	2014	Minimally invasive surgery
Momentis surgical	Israel	2013	Gynecologic surgery
Neocis Inc	United States	2009	Dental implants
QUANTUM surgical	France	2017	Hepatology, oncology
Remebot	China	2010	Neurosurgery
Renishaw plc	United Kingdom	1973	Neurosurgery
Restoration Robotics Inc	United States	2002	Hair restoration
Rob Surgical Systems	Spain	2012	Laparoscopy
Robocath	France	2009	Cardiovascular surgery
RoboticScope	Austria	2017	Visualization system
Smith & Nephew PLC	United Kingdom	1856	Orthopedic surgery
Stryker Corporation	United States	1941	Orthopedic surgery
SurgiScope	France	1989	Neurosurgery
Think Surgical Inc.	United States	2007	Orthopedic surgery
Titan Medical Inc.	Canada	2008	Minimally invasive surgery
Vicarious Surgical Inc.	United States	2015	Minimally invasive surgery
Virtual Incision Corporation	United States	2014	Laparoscopy
WEGO	China	1988	Laparoscopy
XACT Robotics Ltd	Israel	2013	Laparoscopy
ZEISS	Germany	1846	Eye surgery
Zimmer Biomet Holdings Inc.	United States	1927	Neurosurgery

**Fig. 1:**
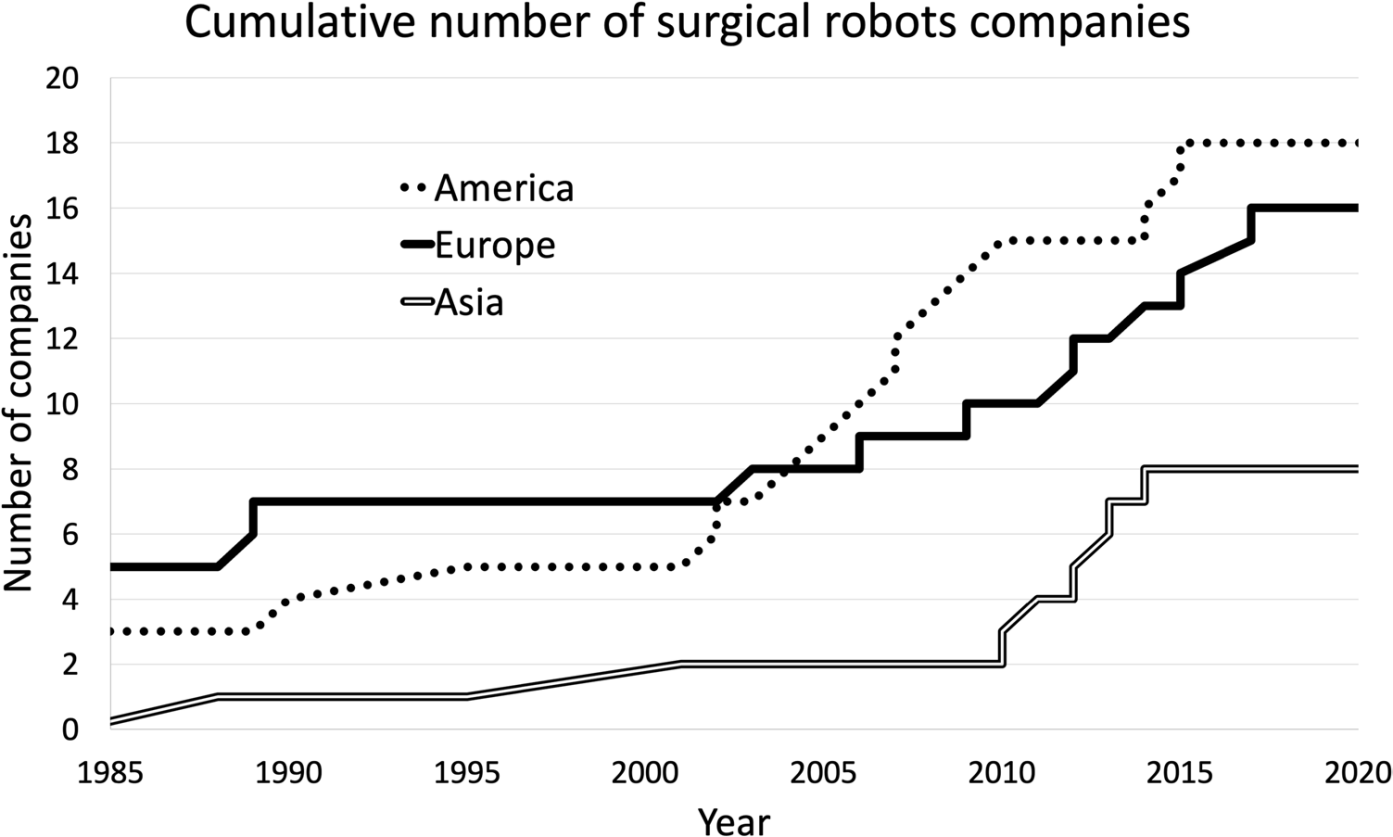
Overview of the topics covered in this survey

The list of surgical robot manufacturers provided, while not exhaustive, allows us to take an outlook of the sector. The United Kingdom has an old tradition of surgical robotics. The United States is one of the most important players. Europe is showing a growing interest in the field. Asia is also following this global innovation trend. The data of Table 1 finds a graphic representation in Fig. 1. A description of each of the selected firms is now provided. Their main commercial products are shortly described.

### Accuray Incorporated

Accuray Incorporated focuses on developing cutting-edge medical technology solutions for the treatment of cancer and other illnesses. The business is headquartered in Sunnyvale, California, and was established in 1990. To provide precise and accurate control during radiation therapy procedures, Accuray has created sophisticated robotic systems. The company’s commercial product line-up includes *CyberKnife* [7], *TomoTherapy* system [8] and *Radixact* Treatment Delivery System [9]. Stereotactic body radiation therapy and stereotactic radiosurgery are two applications for *CyberKnife. The* system minimizes damage to surrounding healthy tissue, by accurately delivering radiation to malignancies, using cutting-edge imaging technology. With the *TomoTherapy* system, treatment regimens may be tailored precisely and individually for image-guided intensity-modulated radiation therapy. The *Radixact* Treatment Distribution System enables precise radiation delivery during treatment. The end effectors made by Accuray are used in radiation treatment procedures in conjunction with their robotic systems. To meet diverse treatment requirements, the end effectors are available in a range of sizes and shapes. Because of its simple interchangeability, procedures can be carried out with more flexibility.

### Asensus surgical

TransEnterix was founded in 2006 in North Carolina. Since February 2021, TransEnterix has changed the name to Asensus Surgical. The international company, formed by over 200 employers, is focused on surgical technology. *Senhance Surgical System* is the main company product. The laparoscopic system features eye-tracking camera control, haptic sensing, improved ergonomics, an open footprint for clear communication and access and reusable instruments. The *Senhance system* is studied to have a similar cost to classic laparoscopy. For example, *Senhance* can be equipped with 3 mm instruments for hernia repair. Pediatric surgical operations are also possible [10]. The robotic platform can be used to make hernia repair, cholecystectomy, gynecologic and colorectal surgery.

### Auris Health Inc.

The company Auris Health Inc., established in California in 2007, produces minimally invasive surgical robots. In 2019 Auris Health was acquired by Ethicon, part of the Johnson & Johnson Medical Devices. The Health *Monarch Platform* [11], is used for therapeutic and diagnostic bronchoscopy treatments. The *Monarch EBUS* [12], an additional product from Auris Health, detects and stages lung cancer. The platform offers advanced vision and fine control. To meet diverse surgical needs, the end effectors come in different diameters.

### AVRA Medical Robotics Inc.

AVRA Medical Robotics Inc. creates surgical robotic systems for minimally invasive treatments. The company is headquartered in Orlando, Florida, and was launched in 2015. The *ARVIS* system, Autonomous Robot for Minimally Invasive Surgery, is the only product sold by AVRA Medical Robotics. The *ARVIS* system [13] is used in laparoscopic surgeries. With the use of proprietary software and cutting-edge imaging and sensing technologies, the system allows the robot to operate on its own, performing surgical tasks without direct human assistance. The *ARVIS* system is equipped with different end effectors. The end effectors are readily replaceable, providing more flexibility during treatments, and they are available in a variety of sizes and forms to meet varied surgical needs. The end effectors have good dexterity and can accurately and precisely execute a broad variety of surgical activities.

### BEC medical

BEC ROBOTICS is a technology company working on human–robot collaboration in the medical, entertainment and industrial sectors. BEC Medical was founded in 2003. The scope of the company is to provide a safe interaction between humans and robots. The company produces *Exacure* and *guidoo. Exacure* is a robot-assisted patient positioning system, specially developed for radiotherapy. The system allows positioning under neutron irradiation in BNCT treatment. *Guidoo* can be used for percutaneous biopsies and ablative procedures. *Guidoo* supports the positioning of needles. The system accuracy reduces the control scans.

### Brainlab

Brainlab is a German company founded in 1989. The company produces *Loop-X™* a fully robotic intraoperative imaging device for surgery. The platform provides the surgeon with high-end intraoperative 2D and 3D imaging. The patient is lying on a robot bed. The system moves the scan area to the region of interest. The patient's body is scanned. Then, using a laser, *Loop-X™* projects both the incision start and end points directly onto the patient’s skin. The system can be used for spinal surgery.

### Cambridge Medical Robotics Ltd. 

Cambridge Medical Robotics Ltd. (CMR) is a UK-based business, established in 2014, that designs and produces minimally invasive surgical robots. *Versius* Surgical Robotic System is the main offering of the company [14]; the system consists of a portable robot suitable for a variety of surgical operations. *Versius* is a modular robotic system that performs minimally invasive surgical procedures using several robotic arms. The system's lightweight robotic arm may be readily moved to various locations to carry out various jobs. Moving the system between operating rooms is simple.

There are several end effectors included with the *Versiu*s Surgical System that can be used for different types of surgical operations. During surgery, the end effectors can be simply replaced.

### Collin medical. 

The origin of the Collin France company dates to 1820. *RobOtol®* is a surgical navigator who received a CE mark in 2016. *RobOtol®* is a mechanical system designed for otologic surgery, offering 7 degrees of freedom: 3 rotations, 3 translations, and one distal movement [15]. The design and ergonomics are adapted to the constraints of the operating room. The system is expected to be used in surgery for otosclerosis, cochlear implants, and drug delivery to the inner ear. The instrument holder arm is designed to assist in the insertion of cochlear implants and allows surgeons to use both hands for endoscopic ear surgery.

### Distalmotion

Distalmotion was founded in 2012 as a spin-off of the Robotics Lab of the Swiss Federal Institute of Technology in Lausanne (EPFL). The company produces the surgical platform *Dexter* suitable for minimally invasive surgery. A wide set of laparoscopic instruments is available: staplers, vessel sealers and clip appliers. *Dexter* adopts single-use instruments, removing the complexity of reprocessing. The system is designed to be easily moved from one operating room to another. The *Dexter* platform is open: surgeons are free to select and use their preferred 3D laparoscopes available in the market.

### EndoControl SAS

Founded in 2006, EndoControl SAS is a medical robotics firm located in France. The business focuses on creating and producing medical robots for endoscopic procedures. Medical robots from EndoControl SAS include wristed tools for robotic-assisted interventions, laparoscopic surgery, and Natural Orifice Transluminal Endoscopic Surgery (NOTES).

Among EndoControl SAS's well-known products are: *ViKY® and JAIMy® system*. *ViKY®* system is a modular robot-assisted platform for laparoscopic procedures, while the *JAIMy® system* [16], is a miniature robot designed for NOTES procedures [17]. A variety of motorized devices are included with the *ViKY®* system, offering a high degree of flexibility and adaptability that may be readily changed out during surgery. The *JAIMy®* system has a robotic arm with a 5 mm diameter and 4 degrees of freedom.

### Corindus Vascular Robotics Inc. 

Corindus Vascular Robotics Inc., founded in 2002 and headquartered in Massachusetts, produces precision vascular robots. The company’s main products are the *CorPath GRX System and the CorPath 200 System* [18]. Both systems are used to perform robotic-assisted vascular surgeries such as Percutaneous Coronary Interventions (PCI). The systems are intended to enhance precision, control, and procedural safety for the operator, while also improving clinical outcomes for the patient. *CorPath* comes with a range of end effectors, including guide catheters, guide wires, and balloon catheters. These end effectors are designed to enable precise control and positioning during procedures, while also providing enhanced visualization and safety features. A wide range of end effectors is available.

### EndoMaster Surgical System

EndoMaster Surgical System is a medical company that develops surgical robots for minimally invasive surgery. EndoMaster Surgical System’s flagship product is the *EndoMaster* Robotic System. It is a robotic surgical platform specially designed for minimally invasive digestive system procedures. The system is composed of a surgeon console, robotic arm, and endoscopic camera. The three *EndoMaster* configurations EASE, FLEX and FLIP are designed for specific surgical procedures [19]. The *EndoMaster* Surgical System is used in endoscopic surgeries, such as gastric bypass, bariatric, and general surgery. The *EndoMaster* end effectors are highly dexterous and enable precise movement in tight spaces.

### Human Xtensions

Human Xtensions is a medical company born in Israel in 2012. The company's main product is *HandX*, a surgical device able to extend the human hand’s reach [20]. The handheld robotics device is an affordable laparoscopic instrument. *HandX* is like a human extension inside the anatomy. The device allows the surgeon to reach ergonomic positioning close to the patients. *HandX* is the pioneer of a new product segment, positioned between traditional laparoscopy and classic robotic surgery.

### Intuitive Surgical Inc.

Intuitive Surgical Inc. was founded in the US in 1995. The company produces *da Vinci* surgical systems. The system is used for the following surgical procedures: urologic, gynecologic, thoracic, cardiac, and colorectal. The Sunnyvale company has offices in Europe and Asia. The *da Vinci* surgical system is made of a surgeon's console, a patient-side cart, and a set of robotic arms. The surgeon, looking inside a high-definition 3D camera, controls the robotic arms. The platform offers vision magnification and translates the surgeon's hand movements into smaller movements of the surgical instruments. *Da Vinci* Surgical System has been released in different versions: Xi, X, SP, and Si [21]. The platform costs around $2,000,000. The end effectors mimic the human wrist. The surgical instruments can cut, grasp, and suture.

### Mazor Robotics Ltd.

Mazor Robotics Ltd. is a medical robotics company founded in 2001 in Israel, which specializes in the development of surgical guidance systems and robotic-assisted surgery. Mazor Robotics has developed three main products for surgical guidance and robotic-assisted surgery. The *Mazor X* [22] is a robotic platform used for spine surgery that provides pre-operative planning, intra-operative guidance, and real-time imaging. The *Renaissance* is a guidance system for the spinal surgery that helps surgeons place screws and implants with high precision [23]. The *Mazor Core* is a module that integrates with the *Mazor X* to provide more accurate and efficient surgical planning. The *Renaissance* Guidance System is used in spine surgeries, such as pedicle screw fixation and interbody fusion. The price is about $ 900,000. *Mazor* end effectors are specifically designed to handle spinal implants, such as screws and rods.

### Medrobotics Corporation 

Medrobotics Corporation Company, founded in 2005 in Massachusetts USA, produces the *Flex®* Robotic System, designed for minimally invasive surgical operations [24]. The *Flex®* Robotic System has a flexible robotic arm that performs transoral procedures in hard-to-reach areas. A high-definition camera allows vision. The range of end effectors includes graspers, scissors, and retractors. The modular end effectors can be easily swapped out during a procedure. *Flex®* Robotic System can be used for tongue resection, polypectomy, and vocal cord procedures.

### Medtronic Inc.

Medtronic Inc., founded in 1949 in Ireland, is a medical company that produces robotic surgical solutions. The company sells globally its products. In December 2018, Medtronic acquired Mazor Robotics for $1.7 billion. The surgeon, thanks to sensors on the end effector, receives real-time feedback, enabling better control. Medtronics produces *Mazor X* Stealth Edition for spine robotics [25], *O-arm* Imaging System, *Midas Rex Legend EHS Stylus* and Medtronic *NIM nerve monitoring system*. Medtronic's surgical robots are designed to work with a variety of end effectors, which are specialized instruments used to perform specific surgical tasks, including electrosurgery, suction and irrigation, grasping, cutting and stapling.

### Meerecompany Inc.

Meerecompany is a medical robotics company founded in 2019 and headquartered in Germany. The company specializes in developing and manufacturing surgical robotic systems that enable precision in minimally invasive surgery. The company’s vision is to make surgery less invasive, more efficient, and patient-friendly. Meerecompany surgical robots have a modular design and can be customized for various surgical applications. They use a haptic feedback system to provide surgeons with tactile sensation and precise control during surgery. The *Meere Surgical System*, from Meerecompany, comprises a surgical console, a robotic arm, and an endoscopic camera [26, 27]. The system suits general, gynecologic, urological, and thoracic procedures. The range of end effectors includes graspers, scissors, dissectors, and suction instruments.

### Microbot Medical Inc.

Microbot Medical Company produces minimally invasive surgery robots. These devices are used for neurosurgery, urology, and gynecology. Some of Microbot Medical Inc.'s commercial products include *TipCAT, LIBERTY™* and *Self-Cleaning Shunt. TipCAT* is a robotic catheter system for the diagnosis and treatment of peripheral arterial disease [28]. *LIBERTY™* is a robotic system for endovascular procedures [29]. *Self-Cleaning Shunt* is a robotic system that treats hydrocephalus [30]. The end effectors can perform aspiration, ablation, and cutting.

### Microsure

Microsure was founded in 2014 in the Netherlands. *MUSA-3* is a microsurgical robot made of a surgeon console and a robotic arm cart [31]. Disposable adapters are used to connect the surgeon's instruments. The *MUSA-3* dexterity enables very precise suturing. The robot can be used for a variety of surgical operations, since it can carry out complex movements on difficult wound surfaces. Using a digital or hybrid microscope, the surgeon views the screen while seated at the surgeon console. Utilizing *MUSA-3* in conjunction with digital microscopes increases the system's ergonomics. The joysticks, which have a large workspace to allow for ample movement, control the robot. To improve accuracy, tremor filtering and movement scaling are applied when transferring joystick movements to the robotic arms.

### MMI

The company MMI was born in Italy in 2015. MMI produces the *Symani®* Surgical System. The robotic platform is suitable for microsurgery and super microsurgery. Two wristed robotic arms, equipped with the NanoWrist® robotic micro instruments, make up the *Symani®* Surgical System [32]. The wrist's seven degrees of freedom provide the control and accuracy required for surgeons to manipulate delicate sutures and procedures. The system features tremor filtration and 7–20 × motion scaling. Surgeons can execute precise surgical procedures and scale their hand movements. The surgeon moves the manipulators directly with the ergonomic *Symani®* Console, just like they would with manual instruments. A heads-up 3D visualization system can be utilized with the console.

### Momentis Surgical

Momentis Surgical was founded in 2013 and is in Israel. The company focuses on creating a surgical robotic system with fingers. Co-founders Dvir Cohen and Nir Shvalb, PhD, have studied how to maneuver instruments within the body, reducing the number of instrument portals needed. The *AnovoTM Surgical System* has arms that replicate the surgeon's shoulder, elbow, and wrist movements [33]. This allows surgeons to use the fundus-to-cervix technique without multiple abdominal incisions. Because the system is less expensive and has a smaller footprint than traditional robotic systems, more hospitals may use it.

### Myomo Inc.

Myomo Inc. Company produces myoelectric orthotics. This technology helps to enhance the mobility of people with difficult neurological conditions like stroke, sclerosis, and spinal cord injuries. The orthotics allow patients to regain mobility. The patient’s muscle signals are amplified and used to control the movement of the device. Myomo Inc. produces *MyoPro* [34] and *MyoCare* [35]. The end effectors can be customized according to the individual patient's needs.

### Neocis Inc.

Neocis Inc. has been founded in Florida in 2014. The company produces robots for dental implants. The *Yomi* Robotic System, from Neocis Inc., provides real-time visualization and guidance for dental implant procedures [36]. *A* robot arm guides the dentist's instruments with high precision, improving accuracy and reducing complications. The end effector is a drill controlled by the dentist by a foot pedal. Haptic feedback, detects the presence of hard or soft tissues, allowing for greater accuracy during the implant placement.

### QUANTUM Surgical

QUANTUM Surgical is a company founded in France in 2017. QUANTUM develops robots for hepatology and oncology. *Epione®* is a robotic-assisted technology for the treatment of tumors. Thanks to a fusion technology, that coordinates the robotic arm and the images, *Epione®* allows to target tumors [37]. The *Epione®* device is CE-marked for abdomen and lung indications, and FDA-cleared for abdominal ablation indication. For example, the platform may be used for minimally invasive liver cancer treatment. First, the tumor is seen in 3D. Then the ablation modality is defined. The robot is registered to the patient synchronizing the breath. The robot reaches the planned trajectory and delivers the ablative therapy. Finally, the CT images are used to assess the results.

### Remebot

Remebot is a robotic company founded in China in 2010. The company produces neurosurgery robots having an accuracy of 0,5 mm and a registration time of under two minutes. The *Remebot neurosurgical robot* is a navigation and orientation robot for neurosurgery [38]. The system integrates image processing and surgical planning, automatic positioning and navigation, and a multi-functional surgical operation platform. It can assist doctors in completing nearly one hundred operations. Some examples are Hematoma Aspiration, Percutaneous Puncture, Craniotomy Navigation and Endoscopic Neurosurgery. Surgery planning software can complete multi-modal fusion including computed tomography (CT) and magnetic resonance imaging (MRI). The optical tracker can automatically identify customized markers, and establish a one-to-one mapping relationship between the three-dimensional model in the software and the real scene to achieve precise navigation and positioning.

### Renishaw plc 

Renishaw plc is a global engineering company born in 1973. The company specializes in the development, manufacture, and sale of precision measurement and control equipment, including metrology systems, spectroscopy systems, and motion control systems. Renishaw designs and manufactures *Neuromate* [39] a surgical robot for neurosurgical procedures. This system can be used for brain biopsies and stimulation. Also, stereotactic neurosurgery can be performed. The surgeon, from a computer console, controls the robotic arm. *Neuromate* surgical robot comes with a range of end effectors that vary in size and dexterity, with some being designed for fine, delicate work and others for larger, more robust tasks. The Renishaw's products also include Coordinate Measuring Machines (CMMs), laser encoders and additive manufacturing systems. Renishaw offers a range of CMMs including: the *Equator™ gaging system*, a versatile gaging system that can be configured to match specific inspection needs the *REVO®* 5-axis scanning system [40] which offers a unique head system that can carry both tactile and non-contact probes. *REVO®* may be used for prostatectomy and cholecystectomy. The company also provides software solutions for data analysis and visualization.

### Restoration Robotics Inc.

Restoration Robotics Inc., recently merged with Venus Concept, is a medical device company focused on developing and commercializing the *ARTAS®* Robotic Hair Restoration System [41]. The *ARTAS®* Robotic Hair Restoration System is a minimally invasive, image-guided robotic system for hair restoration procedures. The system uses advanced algorithms to identify and select the optimal hair follicles for harvesting and then performs follicular unit extraction (FUE) using robotic technology. The system is designed to improve the precision, consistency, and speed of hair restoration procedures while reducing the trauma and scarring associated with traditional manual methods. The *ARTAS*® Robotic Hair Restoration System uses a small, motorized tool called a punch to extract individual hair follicles from the scalp. The punch has a diameter of 0.9 mm, which is smaller than traditional manual punches, allowing for more precise and accurate extraction. The system also includes a set of needles that are used to create the recipient sites for the transplanted follicles. The needles are adjustable in size and depth, allowing for customizable hair restoration procedures.

### Robocath

Robocath company was founded in 2009 by Philippe Bencteux. The company produces smart robotic solutions to treat cardiovascular diseases. The robotic approach maximizes the security of coronary angioplasty using robotic assistance. The goal of this medical operation is to revascularize the heart muscle by putting one or more stents into the blood vessels that supply it. The medical robot *R-One* + *™* comprises a Command and a Robotic Unit [42]. The medical robot has an articulated support and can use guidewires, balloons, or stents.

### RoboticScope

BHS was founded in 2017 in Innsbruck, Austria. The company produces microsurgery devices. *RoboticScope®* is a microscopic visualization system that uses a head-mounted display (HMD), a robotic arm, and a complete digital camera system to replace the conventional setup of fixed eyepieces and microscopes [43]. The HMD's two digital micro screens provide the surgeon with real-time, three-dimensional (3D) video of the surgical site, sourced from the robotic camera unit. With simple head movements, the surgeons control the camera while maintaining a constant gaze on the surgical field and without taking their hands off it.

### Rob Surgical Systems Inc.

The Rob Surgical spin-off was founded in Barcelona in 2012. The Institute for Bioengineering of Catalonia (IBEC) and the Polytechnic University of Catalonia (UPC) founded Rob Surgical in response to the European Robotic Surgery Project (EuRoSurge), which was funded by the European Commission in the FP7, and the Mayo Clinic in the United States regarding minimally invasive robotic surgery (MIRS). The company’s goal is to make surgical robots more effective so that they can replace more conventional surgical techniques. The company has two products: the *Bitrack System* and *Hybrid Surgery* [44]. *Bitrack System* performs abdominal and pelvic surgical procedures. *Hybrid surgery* allows robotic and traditional lap instruments to operate simultaneously.

### Smith & Nephew PLC

Smith & Nephew PLC is a British medical technology company that produces various medical devices, including joint replacement systems, sports medicine implants, wound care dressings, and trauma devices.

Some of the product lines by Smith & Nephew include *JOURNEY II* Total Knee System, *POLAR3* Total Hip Solution, *NAVIO* Surgical System, *HEALICOIL PK* Suture Anchor, *PICO* Single Use Negative Pressure Wound Therapy System, and *DYONICS POWER II* Control System [45]. Cutting, coagulation, and suturing end effectors are controlled by surgeons. In February 2022 the company Smith & Nephew announced the launch of the *CORI* robotic-assisted surgical system for knee arthroplasty [46]. The compact robotic solution includes 3-D intraoperative imaging with a robotic milling tool.

### Stryker Corporation

The Stryker Corporation, founded in 1941 in Michigan produces medical equipment, such as implants, surgical instruments, endoscopy equipment, and neurotechnology products. Some of the company's products are *Mako Robotic-Arm* Assisted Technology [47], *Neptune Waste Management System* [48] and *SurgiCount Safety-Sponge System* [49]. *Mako* system is suitable for orthopedic surgeries, and joint replacement surgeries, such as hip and knee replacements. The *Mako* system costs $1.25 million for the robot, and an added $100,000 service contract is needed every year. Stryker Corporation's Mako Robotic-Arm Assisted Technology is equipped with end effectors that have high precision and accuracy.

### SurgiScope

In 1989, the University of Grenoble and the business AID started developing SurgiScope (ISIS Robotics, Saint Martin d'Hères, France). At least forty *SurgiScope* robots have been sold and installed. Based on a parallel delta mechanism, this ceiling-mounted 7 DoF robotized manipulator is primarily used for neuro-navigation applications or endoscopy and biopsy procedures [50]. SurgiScope can mount a wide range of end effectors.

### Think Surgical Inc.

Think Surgical, founded in 2006, is a California-based medical technology company that develops and manufactures surgical robots and automation technology for orthopedic surgery. Think Surgical primary product is the *TSolution One Surgical System*, a robotic platform designed to assist surgeons in total hip arthroplasty procedures [51]. The system includes a 3D preoperative planning workstation that enables surgeons to plan and simulate the surgery in a virtual environment, as well as a robotic arm that assists in the preparation of the hip socket and placement of the implant.

The *TSolution One Surgical System* uses a range of surgical instruments and end effectors, including bone mills, broaches, rasps, and reamers, which are used to prepare the bone and place the hip implant. The system's end effectors are designed to provide precise control and accuracy during surgery, while also reducing the physical strain on the surgeon. *TPLAN* is a software platform developed by Think Surgical that helps surgeons construct customized 3D surgical plans from CT images of patients. The pre-operative plan, generated by the surgeon in the 3D Planning Workstation, is utilized by the computer-assisted *TCAT* equipment to prepare the joint surface and bone cavity.

### Titan Medical Inc.

Titan Medical Inc. Company, founded in 2008 in Canada, produces robotic surgical technologies. The company produces the *Sport* robotic surgical system, a versatile minimally invasive surgical platform suitable for different surgical procedures. In 2020, Titan rebranded its Sport surgical system to the *Enos robotic single-access surgical system*. The surgeon, thanks to the 3D view, controls multi-articulating instruments. The single-port robot can be used for urology, gynaecology and general surgery [52]. The end effectors are multi-articulated. For example, *Sport* can be used, for example for cholecystectomy and prostatectomy.

### Vicarious Surgical Inc.

Founded in 2014, Vicarious Surgical 

### Virtual Incision Corporation

Virtual Incision Corporation, founded in 2006 produces surgical robotic devices. The company robot *MIRA (Miniature *In Vivo* Robotic Assistant)* allows abdominal surgeries using a single, small incision in the patient's belly button [53]. *MIRA* allows the execution of the following procedures cholecystectomy, colorectal surgery, and appendectomy. The robot's arms can use graspers, scissors, and cautery devices. The surgical instruments are designed to be disposable, reducing the risk of infection and minimizing the need for sterilization. *MIRA*'s end effectors wish to replicate the same degree of precision and control as traditional laparoscopic instruments.

### WEGO

WEGO, a Chinese enterprise founded in 1988, is the manufacturer of the *MicroHand* S Surgical Robot System [54]. This apparatus is a minimally invasive surgical robot designed for laparoscopic surgeries. Its robotic arm is flexible enough to execute 540-degree rotations at the end, accurately replicating the surgeon’s hand movements. Additionally, the robot swiftly and automatically stitches wounds and carries out other basic medical activities. A dual CMOS sensor 3D stereoscopic imaging system installed on the endoscope produces high-resolution, real-time images.

A study has compared the *MicroHand* S and the *da Vinci* surgical robot [55]. The *MicroHand S* robot is slower to install respect to the *da Vinci* robot. *MicroHand* S offers lower total hospital and surgery costs. The operation time is similar.

### XACT Robotics Ltd.

In 2013 was found in Israel the XACT Robotics Ltd. Company. *XACT* Robotic System is a minimally invasive surgical robot featuring high accuracy [56]. A miniature robot navigates inside the body’s natural pathways. The robot uses small and flexible reusable end effectors. Sensors provide real-time feedback. The robot can be used for the following applications: ablation, biopsy, drainage, coil markers, brachytherapy, pain management and drug delivery.

### ZEISS

ZEISS Company was founded in Germany in 1846. The company has developed *Preceyes* a robot assistant for retina surgery [57]. The has a precision of 20 μm. This high surgical precision improves treatment outcomes. The System is compatible with a wide range of instruments. Anesthesia, either local or general, is required for patients. Scaling of gestures and filtering of hand tremors allow to increase the surgery precision. The PRECEYES Surgical System has been clinically validated, received a CE mark and is commercially available.

### Zimmer Biomet Holdings Inc.

Zimmer Biomet Holdings Inc. is a global leader in musculoskeletal healthcare that designs, develops, manufactures, and markets orthopedic reconstructive products, spine and trauma devices, dental implants, and related surgical products.

Zimmer Biomet offers a wide range of medical devices and surgical products, including joint replacement and reconstruction products, spine and dental implants and surgical instruments.

The *ROSA ONE® system*, from Zimmer Biomet, is used for neurosurgery (such as brain biopsies and electrode placements) and spine surgery [58]. *ROSA ONE®,* for example, allows pedicle screw fixation and interbody fusion. Some of the notable product names by Zimmer Biomet include the *Persona Knee System*, the *Mobi-C Cervical Disc*, the *Vanguard 360* Revision Knee System, the Comprehensive *Reverse Shoulder System*, the Comprehensive *Segmental Revision System,* and the *Taperloc Complete Hip Stem*.

Zimmer Biomet's surgical instruments and end effectors are designed to help surgeons perform complex procedures with greater precision and accuracy. The company produces different surgical instruments. Some examples are scissors, needle holders, retractors, and bone-cutting tools.

### Comparison of robotic platforms and end effectors

All the robotic end effectors enable the precision execution of procedures. Each end effector is designed for specific procedures. For example, the *Stryker Mako system* is specifically designed for joint replacement procedures, while the *Smith & Nephew NAVIO system* is designed for both joint replacement and spine procedures. The surgical procedures performed by some of the main robot end effectors are briefly discussed, in Tab. 2.

**Table 2:** Common procedures of robotic platforms

End-effector	Type of surgical procedure
Intuitive Surgical, EndoWrist	General surgery, urology, gynecology
Medtronic, StealthStation S7	Neurosurgery, spine surgery
Stryker, Mako System	Orthopedic surgery
Asensus Surgical	General surgery, gynecology
Zimmer Biomet ROSA Knee	Knee surgery
Smith & Nephew, NAVIO	Orthopedic surgery
CMR Surgical, Versius	General surgery
Titan Medical, SPORT	General surgery

Depending on the system and the kind of work being done, robotic surgical systems have varying degrees of accuracy.

Designed for joint replacement surgery, the *Mako* Robotic-Arm Assisted Technology makes use of a 3D model of the patient's anatomy to help plan and carry out the process with extreme precision, leading to better alignment and positioning of the implants.

The *ROSA ONE® Surgical Robot* offers precise and accurate targeting of surgical tools and implant placement. It is used in neurosurgery and spine surgery. Comparably, it has been demonstrated that the *ExcelsiusGPS system*, which is used in spine surgery, can precisely and accurately target pedicle screws and other spinal instrumentation. Robotic surgical systems are extremely accurate and precise, which can result in better surgical results and quicker patient recovery times. The end effectors range of motion varies greatly depending on the surgical instrument, Tab. 3:

**Table 3:** Mobility of some popular End-effectors

End-effector	Range of Motion (degrees)
Intuitive Surgical, EndoWrist	Up to 540 degrees
Stryker, Mako System	Limited
Asensus Surgical, Senhance	Up to 360 degrees
Zimmer Biomet ROSA Knee	Limited
Smith & Nephew, NAVIO	Up to 360 degrees
CMR Surgical, Versius	Up to 360 degrees
Titan Medical, SPORT	Extended

The offer of robotic devices is wide. Each surgeon is interested and specializes in a specific set of procedures. Table 4 has been created to easily find the commercial products that can be used to perform similar procedures.

**Table 4:** Commercial products sorted by surgical procedures

Procedures	Product	Company name
Bronchoscopy treatments	Health Monarch	Auris Health Inc.
Dental implants	Yomi	Neocis Inc.
ENDOSCOPY: natural orifice transluminal	JAIMy®	EndoControl SAS
Eye surgery	Preceyes	ZEISS
Hair restoration: follicular transplant	ARTAS	Restoration Robotics Inc.
Laparoscopy: abdomen and pelvic	Bitrack System	Rob Surgical Systems Inc.
Laparoscopy: abdomen and pelvic	Hybrid Surgery	Rob Surgical Systems Inc.
Laparoscopy: ablation, biopsy, drug delivery	XACT	XACT Robotics Ltd
Laparoscopy: appendectomy, colorectal	MIRA	Virtual Incision Corporation
Laparoscopy: cardiovascular	R-One	Robocath
Laparoscopy: gastric bypass, bariatric	EndoMaster	EndoMaster Surgical System
Laparoscopy: gynecology, hernia, cholecystectomy	Senhance	Asensus Surgical
Laparoscopy: gynecology, urology, thoracic	Meere Surgical	Meerecompany Inc.
Laparoscopy: gynecology, urology, general, colorectal	Enos	Titan Medical Inc.
Laparoscopy: gynecology, urology, thoracic	da Vinci	Intuitive Surgical Inc.
Laparoscopy: gynecology, hysterectomy	AnovoTM	Momentis surgical
Laparoscopy: gynecologic, urologic, thoracic	MicroHand S	WEGO
Laparoscopy: transoral, colorectal	Flex® Robotic	Medrobotics Corporation
Laparoscopy: hysterectomy and gall bladder	Versius	Cambridge Medical Robotics
Laparoscopy: neurosurgery, orthopedic, microsurgery	Symani	MMI
Laparoscopy: handheld laparoscopy	HandX	Human Xtensions
Laparoscopy	Dexter	Distalmotion
Laparoscopy	MUSA-3	Microsure
Neurosurgery: navigation, endoscopy, biopsy	SurgiScope	SurgiScope
Neurosurgery: treat hydrocephalus	Self-Cleaning Shunt	Microbot Medical Inc.
Neurosurgery	LIBERTY™	Microbot Medical Inc.
Neurosurgery	Neuromate	Renishaw plc
Neurosurgery	Remebot	Remebot
Neurosurgery	ROSA ONE®	Zimmer Biomet Holdings Inc
Oncology: radiotherapy, biopsies, ablative operations	Guidoo	BEC Medical
Oncology: lung cancer detection	Monarch EBUS	Auris Health Inc.
Oncology: radiation	TomoTherapy	Accuray Incorporated
Oncology: radiation, prostate, stereotactic radiosurgery	CyberKnife	Accuray Incorporated
Oncology: treatment delivery	Radixact system	Accuray Incorporated
Oncology, hepatology	Epione	QUANTUM Surgical
Orthopedic: knee arthroplasty	CORI	Smith & Nephew PLC
Orthopedic: hip and knee replacements	Mako	Stryker Corporation
Orthopedic: total hip and knee arthroplasty	TSolution	Think Surgical Inc.
Orthotics: myoelectric orthotics	MyoPro, MyoCare	Myomo Inc.
Otologic: otosclerosis, cochlear implants, drug delivery	Collin medical	RobOtol
Spine surgery: image-guided surgery	Loop X	Brainlab
Spine surgery	Mazor X	Medtronic Inc.
Spine surgery	ROSA ONE®	Zimmer Biomet Holdings Inc.
Vascular surgery	CorPath GRX	Corindus Vascular Robotics Inc.

### Reusable, disposable and reposable end effectors

Surgical robotic systems employ various end effectors to carry out procedures. These end effectors can be reusable, disposable, or deposable. Disposable end effectors are designed for one-time or temporary use. Reposable laparoscopic instruments are formed by reusable and disposable elements: for example, a sterilized handle can be reused, while the interchangeable tips, like scissors, are disposed of. Each family of end effectors can be made of different materials. Unlike disposable instruments, reusable end effectors can be used several times. Stainless steel 316L or titanium are examples of materials used for reusable tools. Disposable tools may be made of 304 stainless. Plastic disposable surgical tools have been successfully produced and tested [59].

Reusable end effectors can be built using titanium or stainless steel, that are resistant to corrosion and degradation. Disposables are often made of 304 stainless steel while reusable of 316L or equivalent.

Disposable tools are relatively cheap, do not need sterilization steps and have limited risk of cross-contamination. Some complex geometries of disposable end effectors are made using 3D printing. Both disposable and reusable effectors may break during surgery. When an end effector is used a limited number of times, environmental issues related to waste management arise. Reusable end effectors usually are made of high-quality durable metals like titanium or stainless steel; these tools are machined with tight tolerances. These end effectors are usually expensive but offer a limited cost per use because can be used even a hundred times. Reusable medical end effectors require cleaning and sterilization procedures for each use. Reusable instruments, that can steer in the body, are often difficult to clean due to the internal driving mechanisms that are based on the cable actuation of the tip. Driven by climate issues, groups are working on fundamentally new steering concepts that should foster the introduction of stronger, thinner, and modular instruments that are driven not by cables but by shaft rotations and translations [60, 61]. It is desirable that hospital policies, in future, will increasingly opt for the use of reusable or recyclable instruments for environmental reasons. Hospital waste management is very complex and heavy to sustain.

## Conclusion

The review has briefly described surgical robots from a selection of companies around the globe. Each robot is designed for performing specific procedures and has varying features and capabilities. Robotic surgery systems offer several advantages over traditional open surgery. Surgical robots can provide surgeons with greater control and precision than human hands, which is essential for delicate procedures. The minimally invasive approach reduces the risk of complications and minimizes the recovery time. Robotic systems provide surgeons with a magnified three-dimensional view of the surgical field, which helps them to better visualize the area. Surgical robots can reduce the surgeon's physical fatigue, as they do not have to hold their arms in one position for long periods. However, robotic surgery also has some limitations. Surgical robots are expensive to purchase, install, and maintain, which can increase the cost of surgical procedures. Robotic surgery requires extensive training for the surgeon and support staff. Only a few robotic surgery systems provide the surgeon with a sense of touch, which can be critical in some procedures [62]. Healthcare professionals, considering incorporating robotic surgery into their practice, can choose from many commercial robots. Market forecasts show, for the future, an increase in the use of service robots [63]. The development of improved user interfaces, powered with artificial intelligence, will enable surgeons to operate more intuitively [64].

Europe has traditionally been an early adopter of robotic surgery technology, with a high concentration of systems per capita. The da Vinci Surgical System by Intuitive Surgical is the most widely used in Europe and has seen significant adoption across the continent. In the United States, robotic surgery systems have also been widely adopted. Other systems, such as the Mako Robotic-Arm Assisted Technology by Stryker and the Rosa Surgical Robot by Zimmer Biomet, have also gained popularity in recent years. In the last years, Asia has shown to be relatively slower in the adoption of robotic surgery systems. Recently countries like China and Japan have increased interest and investment in robotic surgery technology. Systems like the Versius Surgical System by CMR Surgical and the Senhance Surgical System by TransEnterix are gaining traction. Overall, the popularity and the adoption of robotic surgery systems vary depending on region and healthcare system.

## Data Availability

No datasets were generated or analysed during the current study.
